# Supplementary material for: Trace Element Concentrations in Degenerative Lumbar Spine Tissues: Insights into Oxidative Stress
Source: Antioxidants (Basel). 2025 Apr 17;14(4):485. doi: 10.3390/antiox14040485 (PMC12024161; doi:10.3390/antiox14040485)
Supplement: Supplementary file 1 [file antioxidants-14-00485-s001.zip › antioxidants-3522676-supplementary.pdf]

Supplementary

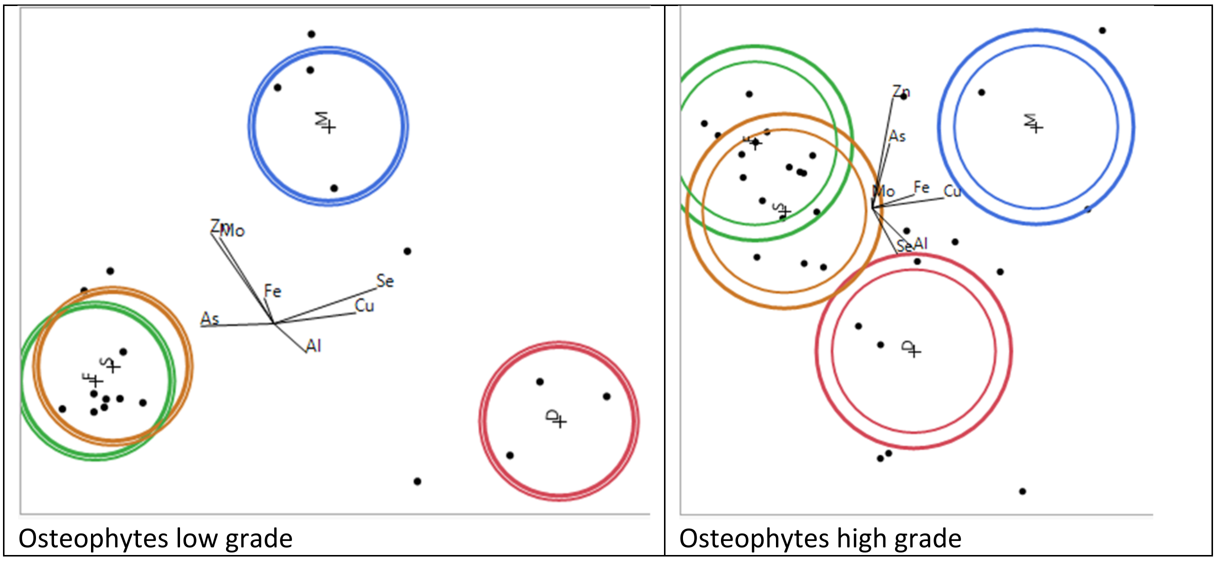

Figure S1. Canonical Discriminant Analysis showing interspecific variation in the concentrations of trace elements in muscle and facet, spinous and intervertebral disk tissues. Ellipses represent the 95% confidence intervals around the group centroid of each tissues in dependence of osteophytes grade

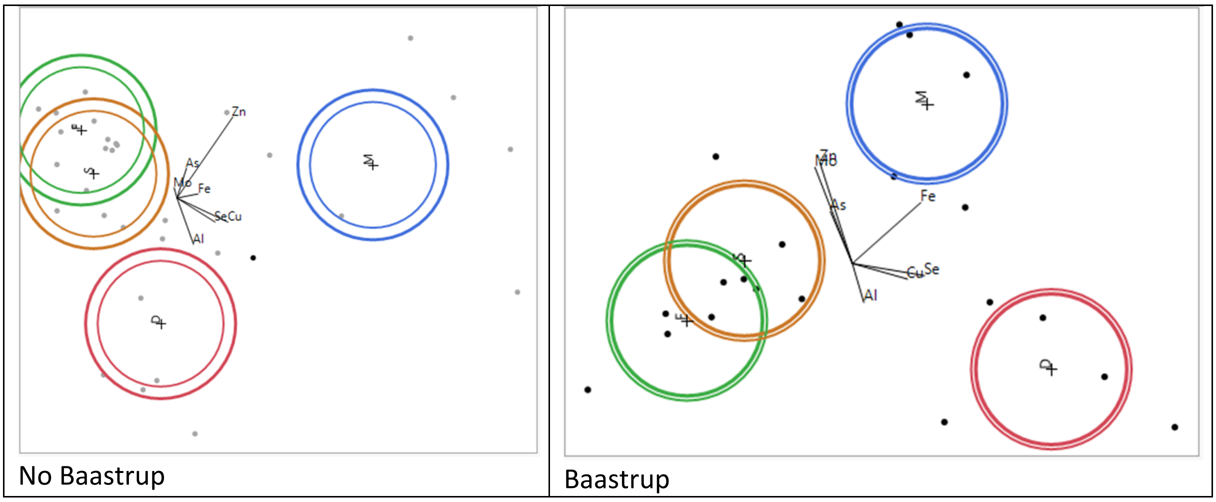

Figure S2. Canonical Discriminant Analysis showing interspecific variation in the concentrations of trace elements in muscle and facet, spinous and intervertebral disk tissues. Ellipses represent the 95% confidence intervals around the group centroid of each tissues in dependence of Baastrup disease

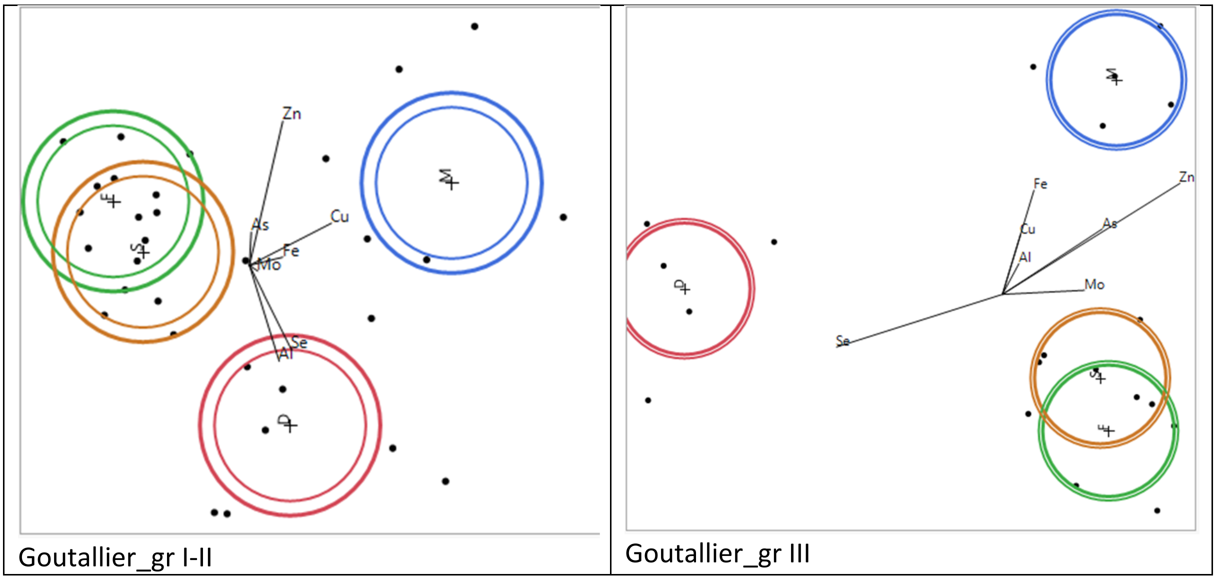

Figure S3. Canonical Discriminant Analysis showing interspecific variation in the concentrations of trace elements in muscle and facet, spinous and intervertebral disk tissues. Ellipses represent the 95% confidence intervals around the group centroid of each tissues in dependence of Goutallier scale

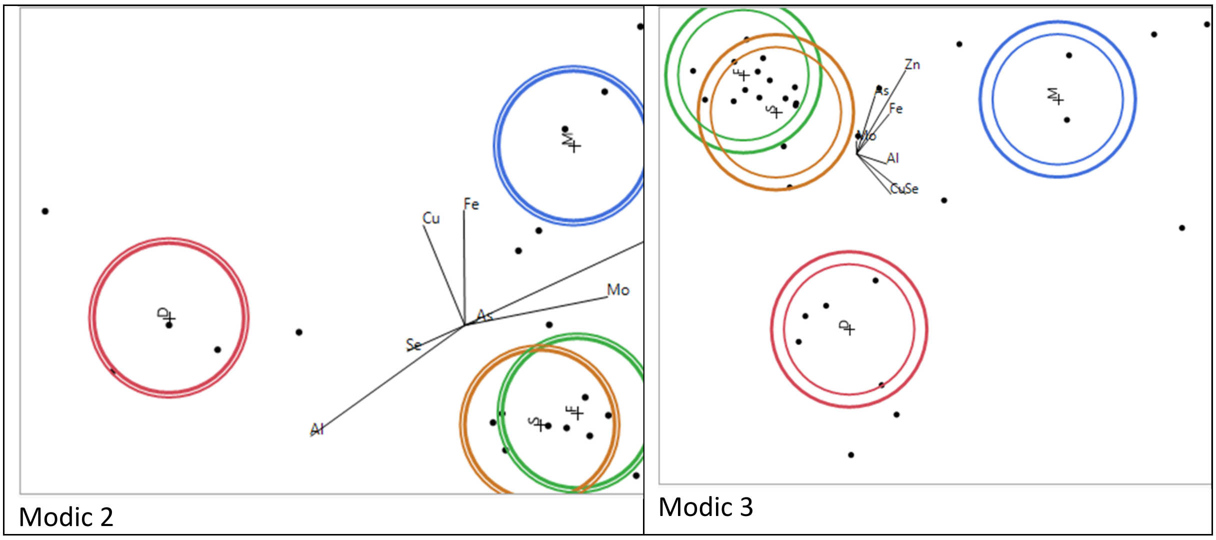

Figure S4. Canonical Discriminant Analysis showing interspecific variation in the concentrations of trace elements in muscle and facet, spinous and intervertebral disk tissues. Ellipses represent the 95% confidence intervals around the group centroid of each tissues in dependence of Modic scale

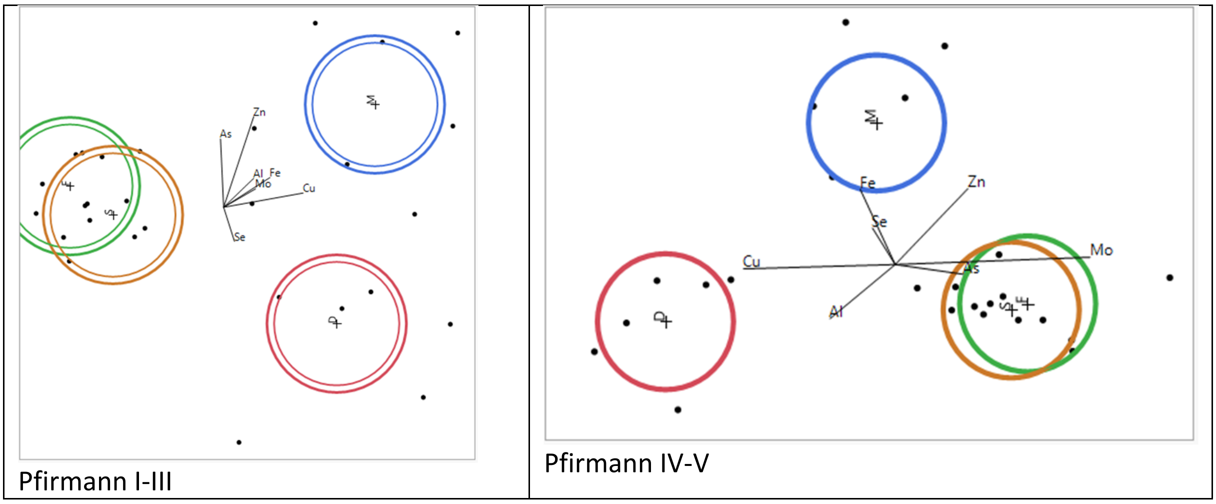

Figure S5. Canonical Discriminant Analysis showing interspecific variation in the concentrations of trace elements in muscle and facet, spinous and intervertebral disk tissues. Ellipses represent the 95% confidence intervals around the group centroid of each tissues in dependence of Pfirmann scale
